# Baculoviruses: Sophisticated Pathogens of Insects

**DOI:** 10.1371/journal.ppat.1003729

**Published:** 2013-11-14

**Authors:** Rollie J. Clem, A. Lorena Passarelli

**Affiliations:** Division of Biology, Kansas State University, Manhattan, Kansas, United States of America; University of Florida, United States of America

The baculoviruses (family: *Baculoviridae*) are a group of large DNA viruses that infect insects. These viruses are well known for their utility and versatility as gene expression vectors, biological pesticides, and vectors for transduction of mammalian cells [1]–[3]. However, baculoviruses are much more than just useful laboratory tools. The rich and fascinating biology associated with these viruses provides many interesting examples of virus-host interactions and virus modification of host processes.

While a few known baculoviruses infect larval mosquitoes or sawflies, the large majority of them infect caterpillars, the larval stages of insects from the order *Lepidoptera* (moths and butterflies). Baculoviruses typically have narrow host ranges, often limited to just one or a few related insect species, although the most intensely studied member of the family, *Autographa californica* multiple nucleopolyhedrovirus (AcMNPV), is able to infect as many as 30 species from several lepidopteran genera. Baculovirus nucleocapsids are rod-shaped and surrounded by an envelope, and they contain circular genomes of double-stranded DNA that range in size from about 80–180 kbp in length. Similar to other large DNA viruses, baculoviruses encode numerous accessory genes with roles in manipulating cellular processes such as the cell cycle and apoptosis [4], [5], as well as host physiology and behavior (see below). However, an unusual feature of baculoviruses is that they produce two distinct types of enveloped virions: occlusion-derived virions (ODV), which are embedded in large (5–10 micron) protein crystals called occlusion bodies and are responsible for horizontal transmission between insects, and budded virions (BV), which spread infection from cell to cell (Figure 1). Furthermore, baculoviruses are the only known nuclear-replicating DNA viruses that encode a DNA-directed RNA polymerase, which is used to transcribe the viral late and very late genes, and is also utilized to express foreign genes in the baculovirus expression vector system. Here, we discuss some recent and exciting developments in the baculovirus field; for a comprehensive review of baculoviruses, see [6].

**Figure 1:**
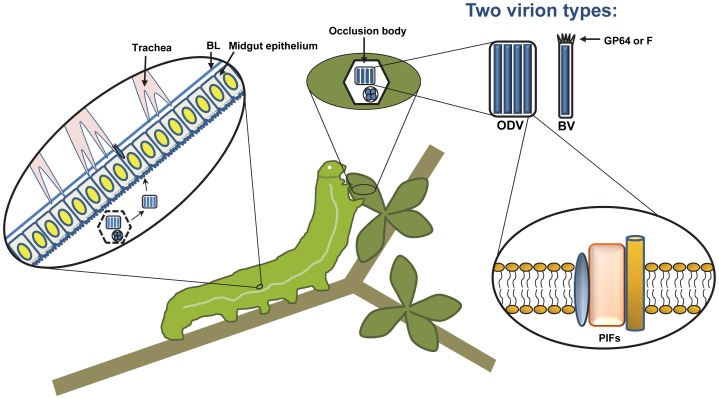
A typical baculovirus replication cycle. Nucleocapsids are produced in the nucleus of infected cells, but two forms of enveloped virions are produced. The first, budded virus (BV), is formed when single nucleocapsids exit the nucleus and bud from the cell, acquiring an envelope from the plasma membrane. The BV attachment and fusion protein, either GP64 or F depending on the type of baculovirus, is concentrated at one end of the virion in structures that are visible by electron microscopy, known as peplomers. Later, the second type of virion, called occlusion-derived virus (ODV), is formed when nucleocapsids obtain an envelope from the inner nuclear membrane. ODV become embedded in large proteinaceous crystals that are primarily composed of a single viral protein called polyhedrin, and are known as occlusion bodies. The occlusion bodies remain within the nucleus until they are liberated by cell lysis. The ODV of some baculoviruses, known as multiple nucleopolyhedroviruses (MNPVs), contain multiple nucleocapsids within a single-enveloped virus particle, as is illustrated here. In nature, the baculovirus replication cycle begins when a susceptible insect larva consumes viral occlusion bodies contaminating their food source. The occlusion bodies dissolve in the highly alkaline environment of the larval midgut, releasing ODV, which attach to the microvillar membranes of midgut epithelial cells. Attachment and fusion occurs via the PIF proteins, found in the ODV envelope. Infected midgut epithelial cells produce BV, which bud from the basal side and infect tracheal epithelial cells. Infection of tracheal cells is thought to be one mechanism that allows BV to escape across the midgut basal lamina (BL) and spread infection throughout the insect. The majority of tissues become infected, producing large amounts of ODV and BV. The infected insects liquefy after death, allowing dispersal of occlusion bodies and promoting subsequent infections.

## 1. Modification of Host Physiology and Behavior

Baculovirus-infected larvae exhibit several interesting examples of pathogen-modified physiology and behavior. One of these involves inhibition of larval development. Normally, lepidopteran larvae pause feeding periodically to go through larval molts. However, baculovirus-infected larvae do not molt, due to the action of a viral enzyme called ecdysteroid UDP-glucosyltransferase (EGT) [7]. EGT prevents larval molting by inactivating ecdysone, the major insect molting hormone. Infected larvae grow larger than normal before succumbing to a dramatic death worthy of a horror film: infected corpses liquefy, due to expression of viral cathepsin-like protease and chitinase enzymes, and liquefaction aids in release of viral occlusion bodies. The amount of progeny virus is impressive, with upwards of 10 million occlusion bodies produced per milligram of larval tissue.

**Figure 2:**
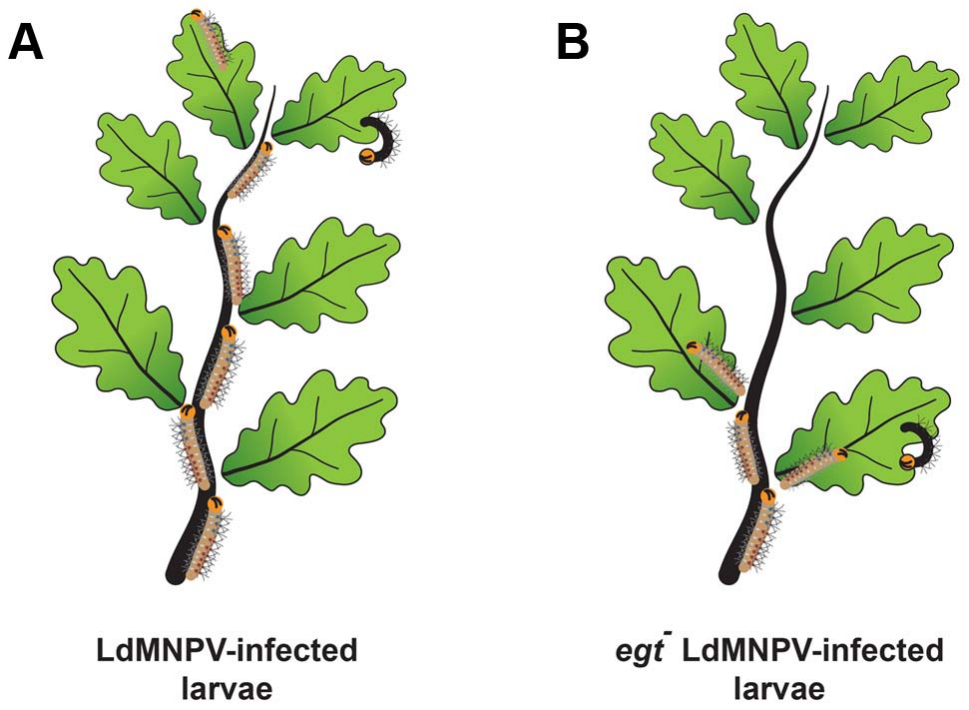
Baculovirus-induced climbing behavior, historically known as “Wipfelkrankheit” (tree top disease), has been attributed to the viral *egt* gene. Gypsy moth (*Lymantria dispar*) larvae that were infected with wild type *L. dispar* MNPV (LdMNPV) (A) demonstrated increased climbing behavior compared to a mutant of LdMNPV lacking the *egt* gene (B) [8]. This evolutionary adaptation is thought to aid in virus dispersal after death of the larvae, resulting in increased dispersal of occluded virus to the rest of the vegetation below.

More recently, it was shown that expression of *egt* by a baculovirus infecting *Lymantria dispar* (gypsy moth) larvae was also responsible for inducing larval climbing behavior [8]. Normally, gypsy moth caterpillars live in the soil and only come out to feed at night, to avoid predation. In contrast, infected larvae climb to the tops of trees and then die, raining occluded virus down on the vegetation below (Figure 2). In yet another example of virus-induced behavior modification, increased larval locomotory activity is induced by expression of a viral protein called protein tyrosine phosphatase (PTP) [9], [10]. Infected larvae exhibit increased wandering behavior, which is also presumed to increase virus transmission. The viral PTP is an active phosphatase and a component of the virion. In one report, the active site of the AcMNPV PTP enzyme was required for the stimulation of larval wandering [9]. However, another report on the *ptp* gene from *Bombyx mori* NPV found that the active site was not required for stimulating wandering, but that the mutant virus lacking *ptp* was less able to infect the brain [10]. Phylogenetic analyses indicate that both the *egt* and *ptp* genes appear to have a lepidopteran origin, as do many other baculovirus accessory genes, indicating that baculoviruses have acquired many advantageous genes during coevolution with their hosts.

## 2. Gut Interactions

Baculoviruses are transmitted orally in nature, and the target of primary infection is the larval midgut epithelium. After being consumed, occlusion bodies dissolve in the midgut lumen, releasing the embedded ODV, which infect midgut epithelial cells. Progeny BV bud from the basal surface of the epithelium, cross the basal lamina (see below), and infect most of the remaining tissues of the larva (Figure 1). The process of ODV attachment and entry depends on several viral proteins found in the ODV envelope, referred to as PIFs (*per os* infectivity factors). Recent results indicate that some of the PIFs form a complex in the ODV envelope, likely interact with an unknown midgut receptor, and mediate fusion with the plasma membrane [11]. Thus the PIFs constitute a novel virus attachment/fusion protein complex. Interestingly, PIFs appear to be ancient genes that are conserved among three related insect virus families, as *pif* homologs are also found in the nudiviruses and the polydnaviruses, the latter of which form mutualistic relationships with parasitic wasps [12], [13].

Once replication has occurred in midgut cells, most baculoviruses (except for a few that only replicate in midgut cells) must escape across the midgut basal lamina, a layer of extracellular matrix, to allow infection of other larval tissues (Figure 1). All of the midgut-escaping baculoviruses sequenced to date encode homologs of fibroblast growth factor (vFGF). The expression of vFGF induces a protease cascade, resulting in degradation of the basal lamina surrounding tracheal cells that service the midgut. Presumably, this allows the virus to infect these tracheal cells and then spread to the rest of the insect [14]. Some baculoviruses may also be able to directly cross the basal lamina of the midgut in certain cases, since virions have sometimes been observed in the hemocoel of larvae shortly after oral infection.

## 3. Regulation of Gene Expression

As with other DNA viruses, baculovirus gene expression occurs as a cascade. Baculovirus gene expression is traditionally divided into three stages, each of which is dependent on expression of the earlier stages: early, late, and very late. The early genes are transcribed by host RNA polymerase II, while late and very late genes are transcribed by the viral RNA polymerase. This unique viral polymerase consists of four subunits that have little homology to other known polymerases. Late and very late promoters are also distinctive and simple, consisting mainly of the tetranucleotide sequence TAAG.

Baculovirus genes are randomly distributed around the circular genome in both DNA strands, with very short intergenic sequences, including many overlapping genes. A recent analysis, using a combination of strand-specific RNA-seq and deep sequencing strategies to comprehensively identify transcription start sites and polyadenylation sites, revealed additional complexity in the transcription pattern of AcMNPV genes [15]. More than 200 transcription start sites were identified, many of which were previously unknown. There are numerous genomic regions where transcripts are derived from both strands; these antisense RNAs may form double-stranded RNA structures and could be involved in gene regulation, either by antisense or RNA interference mechanisms. Indeed, hot spots of siRNAs from a baculovirus genome have been reported [16]. By 12 hours post infection, viral transcripts comprised 38% of the total cellular mRNA, and by 48 hours, a single viral transcript, polyhedrin, was 24% of the total mRNA in the cell. This is consistent with previous observations, including estimates that the polyhedrin protein (the major constituent of occlusion bodies) constitutes 25% of the total protein in the infected cell at this time, and explains why the polyhedrin promoter is heavily utilized in the baculovirus expression system. In addition, 12 spliced mRNAs were identified; only one of these had been identified previously.

An emerging theme in regulation of gene expression is regulation by miRNAs, several of which have been found in baculoviruses [17]. One of these inhibits transport of small RNAs from the nucleus, suggesting active suppression of the cellular miRNA pathway [18].

Another interesting potential mechanism of baculovirus gene regulation involves the presence of specific mixtures of full-length and defective virus genomes in natural virus populations [19]. Optimal infectivity requires a precise molar ratio between different genomes carrying various deletions, and natural selection maintains this ideal ratio. It is thought that this may optimize the expression of certain viral genes that are required for infectivity.

## 4. Viral Proteins Affecting Nucleocapsid Assembly

Bacmid technology, which allows baculovirus genomes to be manipulated in bacteria, has provided an important tool to study baculovirus gene function. Investigators have used bacmids to delete many baculovirus genes, especially the so-called core genes (those genes, currently numbering 37, that are conserved in all known baculovirus genomes), which are often essential for replication. While these include the *pifs* and genes involved in viral DNA replication and late gene transcription, a large number of core genes appear to be required for proper nucleocapsid assembly, even though they are not necessarily structural genes (reviewed in [6], [20]). It is not clear why so many genes are required for virion assembly, but some of them are likely involved in processing and packaging of the DNA genome. Further characterization of these genes will define their specific functions in virion assembly or a process prior to assembly that manifests as a defect in virion morphology.

## 5. Future Research

While we have learned a great deal about baculoviruses over the past several decades, there is still much we do not know. It is likely that we have only uncovered the tip of the iceberg in terms of the diversity of baculoviruses in nature, especially those that infect nonlepidopteran insects. Also, even in the AcMNPV genome, roughly half of the genes have no assigned function, meaning that there are probably many more interesting baculovirus accessory genes whose functions are waiting to be discovered. Since BV can bind to and enter many kinds of vertebrate and invertebrate cells, baculovirus host range is more complex than just receptor binding, and the determinants of host range are poorly characterized. Host range manipulation will be important in developing improved biopesticides and gene therapy vectors. Understanding and manipulating baculovirus-host interactions will be facilitated by deriving additional lepidopteran genomic sequences and tools to genetically manipulate these insects. In addition to the PIFs found in ODV, two types of attachment/fusion proteins (F and GP64) are present in BV, and while these have been fairly well studied, host receptor molecules have not been identified for either ODV or BV. The mechanisms of baculovirus DNA replication and capsid assembly are also still largely undefined. Finally, invertebrate antiviral immunity is still a young field, and little is known about immune responses of insects against baculoviruses. Further research will continue to contribute to our general understanding of virus-host interactions, as well as continue to improve the utility of baculoviruses in biotechnology and agriculture.
